# Potential roles of the prokineticins in reproduction

**DOI:** 10.1016/j.tem.2006.12.002

**Published:** 2007-03

**Authors:** David Maldonado-Pérez, Jemma Evans, Fiona Denison, Robert P. Millar, Henry N. Jabbour

**Affiliations:** 1 MRC Human Reproductive Sciences Unit, The Queen's Medical Research Institute, 47 Little France Crescent, Edinburgh, EH16 4TJ, UK; 2 Reproductive and Developmental Sciences, Centre for Reproductive Biology, The Queen's Medical Research Institute, 47 Little France Crescent, Edinburgh, EH16 4TJ, UK

## Abstract

Prokineticins are multifunctional secreted proteins that were originally identified as regulators of intestinal contraction but subsequently shown to affect vascular function, hyperalgesia, spermatogenesis, neuronal survival, circadian rhythm, nociception, feeding behaviour, immune responses, haematopoiesis and the development of the olfactory and gonadotropin-releasing hormone systems. Their role in the reproductive tract is still not fully elucidated, although they are reputed to increase microvascular permeability. Expression of prokineticins and their receptors has been reported in the ovary, uterus, placenta, testis and prostate. Their expression has also been reported in various pathologies of the reproductive tract, and future studies will highlight whether inhibition of prokineticin function in these pathologies would be a useful therapeutic target.

## Introduction

Prokineticins (PKs) are factors that have been recently described and are involved in a wide array of functions in different tissues through the activation of their cognate receptors. Here, we first give a general background of the structure and functions of PKs and then focus on the role of PKs in reproduction.

## PKs and their receptors

The PKs are two recently identified proteins with roles in physiological and pathological conditions. The names PK1 and PK2 were assigned to these proteins by Li *et al.* [1] to reflect their functions in inducing specific and potent contractions on the smooth muscle of the gastrointestinal tract. Subsequently, LeCouter *et al.* [2] described a growth factor which induced a strong and reproducible mitogenic response in endocrine gland-derived endothelial cells. The similar effects induced by this protein and by vascular endothelial growth factor (VEGF) led it to be named endocrine gland VEGF (EG-VEGF). Although there are several similarities in the functions and control mechanisms of VEGF and EG-VEGF, the two factors are structurally unrelated. The amino acid sequences for PK1 and EG-VEGF are identical, and, for the purposes of this review, the term ‘PK1’ is used throughout.

**Figure 1:**
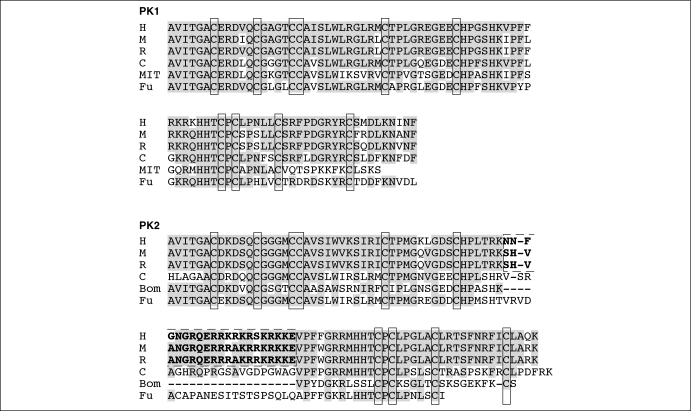
Amino acid sequence comparison between human PK1 and PK2, and several putative orthologues. High sequence homology (shaded grey residues) among species is observed. Ten highly conserved cysteines predicted to form five disulfide bonds are highlighted in solid boxes. In PK2, the residues highlighted in a dashed box correspond to the extra amino acids present in a splice variant that results from alternative splicing of exon 3. Key: H, human (*Homo sapiens*); M, mouse (*Mus musculus*); R, rat (*Rattus norvegicus*); C, chicken (*Gallus gallus*); MIT, black mamba (*Dendroaspis polylepis*); Fu, fugu (*Takifugu rubripes*); Bom, toad (*Bombina variegata*).

PK1 is the human orthologue of a nontoxic protein isolated from the venom of the black mamba (*Dendroaspis polylepis*) and named venom protein A (VPRA) [3] or mamba intestinal toxin 1 (MIT1), owing to its ability to contract guinea pig ileum [4,5]. PK1 is 80% homologous to VPRA/MIT1 and is a mature protein of 86 amino acids, with a signal peptide of 19 amino acids. The gene that encodes the PK1 precursor is located on human chromosome 1p21 [6] and is encoded by three exons [1,2] (Figure 1).

PK2 is the human paralogue of PK1 and orthologue of a protein isolated from skin secretions of the toad *Bombina variegata*, known as Bv8 [7]. A splice variant of PK2 has been described in human, mouse and bull testis, having an arginine and lysine-rich insert of 21 amino acids after residue 47. This led to the splice variant being called ‘Bv8-basic’ (Bv8-b) [8]. The PK2 precursor is located on human chromosome 3p21.1 [6] and is encoded by four exons, with the third exon being subject to alternative splicing [9] (Figure 1).

PK1 and PK2 share ∼44% amino acid identity and also share a common protein structure motif. They have a conserved N-terminal sequence (AVITGA), which is essential for the activity of these proteins. Mutations to this sequence, by insertion of a methionine preceding the N-terminal alanine, substitution of the N-terminal alanine with a methionine [10] or deletion of the first two amino acids [11], produce PK receptor (PKR) antagonists. Another feature of the PKs is the presence of ten conserved cysteines, which are predicted to form five disulfide bonds [1,12] (Figure 1). These disulfide bonds are predicted to form a fold in the PK1 molecule similar to that formed in the related proteins colipase, and Dickkopfs [12,13], which is essential to activity because incorrect folding of recombinant proteins, mutation of the cysteines or substitution of the cysteine-rich domain produces proteins that have no activity at PKRs [10].

**Figure 2:**
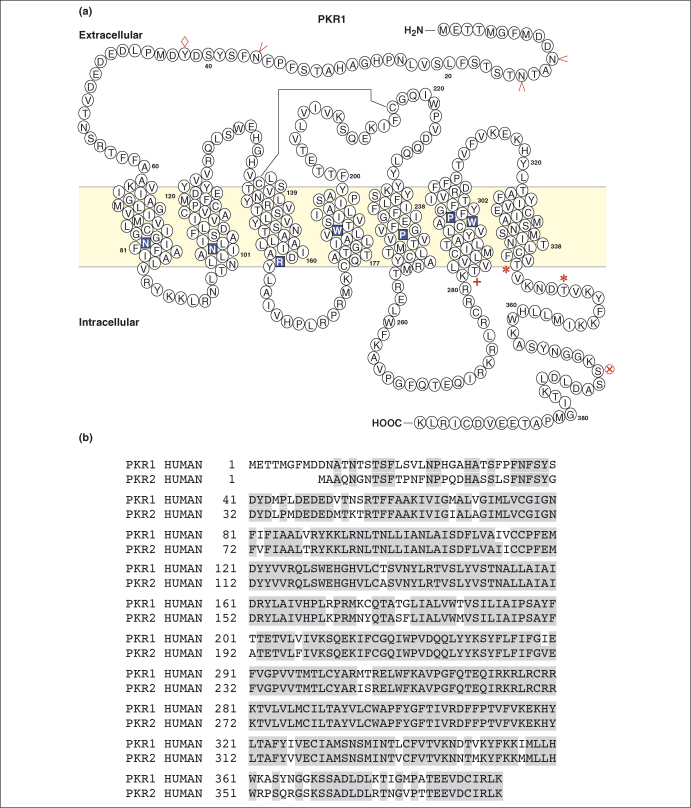
**(a)** Schematic representation of human PKR1. Putative N-glycosylation (<), sulfation (◇) and a disulfide bridge (_∏_) sites are indicated. Serine or threonine residues occurring in putative protein kinase C (∗), protein kinase A (+) and casein kinase II (⊗) phosphorylation sites are also indicated. Residues in blue squares are the ones highly conserved throughout the rhodopsin family of GPCRs. The highly conserved NPXXY motif in the seventh transmembrane domain of the rhodopsin-like GPCR family is changed to NTXXF in PKRs; this could be relevant in terms of the dynamics of receptor internalization [52,53]. **(b)** Sequence alignment for human PKR1 and PKR2. These receptors have 85% homology (depicted by shaded grey amino acids), with the greatest differences in their N-terminal sequences.

The PKs are the cognate ligands for two closely related G-protein-coupled receptors (GPCRs), termed PKR1 and PKR2, which share 85% amino acid identity and exhibit the greatest differences in their N-terminal sequences [14,15] (Figure 2). Their sequences are almost identical in the transmembrane domains [16], suggesting that their activation mechanisms are identical and that small-molecule analogues will not discriminate between the receptors, as is the case for PK1 and PK2. The affinity of these factors for their receptors is similar, with PK2 showing a moderately higher affinity for both receptors (Table 1).

PKRs have been reported to couple either to Gi [17] or to Gq [14,16] proteins. In adrenal cortex capillary endothelial (ACE) cells, activation of the receptors has been shown to be inhibited by pertussis toxin [17], suggesting Gi coupling. By contrast, in transfected CHO cells activation of the receptors has been shown to induce calcium mobilization and phosphoinositide hydrolysis [14–16], suggesting Gq coupling. Signalling through these receptors is linked to phospholipase Cβ activation and generation of diacylglycerol and inositol phosphate, with potential downstream activation of protein kinase C, extracellular-signal-regulated kinases (ERK) 1 and 2, Akt and nitric oxide synthase [14,17,18]. Activation of ERK 1 and 2 through PK1–PKR1 binding has been demonstrated, by use of specific inhibitors, to be important in PK1-induced proliferation and migration of ACE cells [17].

## Functions of PKs and their receptors

PKs were initially reported to be expressed in the gastrointestinal tract, where they were shown directly to stimulate contraction of the ileum longitudinal muscle of guinea pigs [1]. However, the opposing effect of relaxation through a nitric oxide-mediated mechanism has recently been reported in the murine proximal colon [18]. In addition, it has been reported that PK2 has no effect on forestomach or colon contraction [19]. These findings suggest that the intracellular milieu in different tissues results in differential coupling and different phenotypic effects.

PKs are also expressed in steroidogenic tissues, including the testis [8,20], ovary [21,22], placenta [2,23] and adrenal glands [17]. In these tissues, it has been shown that PKs are involved in survival, proliferation, differentiation and induction of fenestrae of capillary endothelial cells [17]. The effect of PKs on endothelial cells seems to be tissue specific. For example, and in contrast to VEGF, PKs have no effect on endothelial cells derived from aorta, umbilical vein or cornea [2].

A role for PKs has also been suggested in haematopoiesis and in regulation of the immune response [24,25]. PK2 is expressed in the bone marrow, as well as in peripheral blood cells, particularly in monocytes, neutrophils and dendritic cells [25], whereas PK1 is expressed in B and T cells, and in inflamed tissues [24]. *In vitro* studies suggest that PKs promote survival and differentiation of the granulocytic and monocytic lineages. Moreover, the expression of PKR1 and PKR2 in progenitor and mature blood cells [25] supports further the proposed participation of PKs in haematopoiesis and the immune response.

PK2 expression has been demonstrated in the central nervous system, and a role in supporting neuronal survival has been suggested [26]. In the suprachiasmatic nucleus (SCN), PK2 exhibits a circadian oscillation profile [27,28]. It has been proposed that PK2 functions as a crucial SCN output molecule responsible for circadian locomotor rhythms [27,29]. PK2 is also expressed in the olfactory bulb, where it is involved in neurogenesis. Studies *in vitro* showed PK2-induced migration of subventricular zone-derived neuronal progenitors [30]. This is supported by mouse knockout studies, in which PK2^−/−^ and PKR2^−/−^ mice showed hypoplasia of the olfactory bulb [28,30]. Interestingly, PKR2 knockout mice also show severe atrophy of the reproductive system, including the testis, ovary, uterus, vagina and mammary gland. Immunohistological studies demonstrated an absence of gonadotropin-releasing hormone (GnRH) neurones in the hypothalamus of these animals, suggesting that activation of PKR2 is required for the correct migration of these neurones from the olfactory placode into the forebrain during development [28]. The PKR2^−/−^ phenotype is strikingly similar to the Kallmann syndrome (KS) in humans, and recently PK2 and PKR2 mutations were identified in several KS patients [31].

PKs are also involved in nociceptive sensitization and regulation of feeding behaviour. Administration of PK2 through several routes decreased the nociceptive threshold to thermal and mechanical pain in rats [32]. In addition, PKR1 knockout mice showed impaired nociceptive and inflammatory pain sensation to noxious heat [33]. Intracerebroventricular delivery of PK2, or its amphibian homologue Bv8, potently suppressed feeding in rats [34].

## The role of PKs and their receptors in human reproductive function

There are two levels at which PKs can modulate reproductive function. As discussed earlier, PKs have an important role in the development of the GnRH system but they can also function directly in reproductive organs.

### Male reproductive function

PKs and their receptors are expressed in the testis and the prostate. In the testis, PK1 is predominantly expressed in testosterone-producing Leydig cells, whereas PK2 is restricted to primary spermatocytes [8,20]. The PKRs are expressed in vascular endothelial cells in the testis [20]. Interestingly, in the mouse testis, PKR1 and PKR2 are expressed equally, whereas in the human testis, PKR1 is expressed at higher levels in comparison to PKR2 [20]. The exact role of each of these receptors and the implications of differential levels of expression remain to be elucidated.

It has been proposed that PKs, through their cognate receptors, function as regulators of proliferation and the formation of fenestrae in the human testis vasculature [20]. As a result, they might contribute to the modulation of the transport of testosterone out of the testis and of regulatory factors into the testis [35]. Additionally, it has been proposed that PKs potentially function as mediators of the inflammatory response during testicular infections [20]. This proposition is based on the fact that the testis is a site of inflammation in response to bacterial and viral infections [36,37], and that PKs have been implicated in the evolution of innate and acquired immune responses [25].

The expression of PKs and their receptors has been reported in the prostate [1,14,38]. However, at the protein level, PK1 expression has been detected only in hyperplastic and cancerous tissue [38]. More studies are necessary to evaluate whether PKs have a role in the normal and diseased prostate.

### Female reproductive function

PKs and their receptors are expressed in the ovary, uterus and in various tissues of pregnancy [21,23,39]. In the normal ovary, PK1 is expressed in a dynamic way in elements of the sex cord–stroma lineage [40], whereas PK2 expression is not detectable [21]. During follicle maturation, PK1 and VEGF expression are inversely related. In primordial and primary follicles, there is high expression of PK1 in granulosa cells but no VEGF expression. Maturing secondary follicles maintain strong PK1 expression and weak to moderate VEGF expression. However, in the antral follicle, PK1 is expressed at low levels in theca cells, whereas VEGF expression is very strong in granulosa cells and moderate in theca cells. In the mature atretic follicle, PK1 expression is strong again in residual theca but VEGF expression is weak [21]. In the corpus luteum, the mRNA expression of PK1 increases as the corpus luteum matures, whereas VEGF expression is already maximal at the early luteal phase [21,22]. These different expression patterns suggest that VEGF and PK1 have different roles in the vasculature and/or non-vascular roles in the corpus luteum. The actions of PK1 in the ovary are likely to be mediated by PKR1 and PKR2, which are expressed in the human ovary [14,16]. However, their precise localization remains to be elucidated.

Studies *in vitro* suggest that PK1 has a role in the proliferation and survival of endothelial cells of the bovine corpus luteum [41]. Also, an indirect role in angiogenesis in the corpus luteum has been suggested following the observation that PK1 can stimulate the expression of VEGF [42].

**Figure 3:**
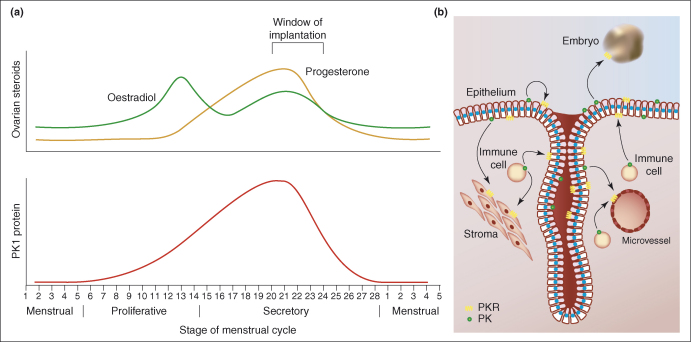
**(a)** Temporal expression of PK1 protein in human endometrium across the menstrual cycle. PK1 protein expression gradually rises from low levels during the early proliferative stage to high levels that peak at the midluteal phase (window of implantation), followed by a decrease in expression in the late luteal phase [43]. This is a result of PK1 being modulated by oestrogen and progesterone in the endometrium [39,43]. **(b)** PKs and their receptors are expressed in different cellular compartments of the uterus. Hence, PKs have the potential to participate in multiple cellular processes. Arrows designate the potential autocrine and paracrine modes of action of PKs through PKRs in the different cellular compartments of the endometrium.

In the non-pregnant uterus, PK1 is expressed in the glandular epithelium, as well as in the endothelial and stromal cells of the endometrium, predominantly in the functional layer [39]. It is also expressed in endothelial cells and smooth muscle cells of the myometrium [39]. PK1 expression is dynamic across the menstrual cycle, with low levels of expression during the early follicular phase, followed by a gradual increase of expression that peaks at the midluteal phase and finally a decrease in expression during the late luteal phase [43] (Figure 3). PK2, PKR1 and PKR2 are also expressed in various cellular compartments of the endometrium but they do not show a temporal variation in their mRNA expression across the menstrual cycle [39]. PK1 is expressed in endometrial tissue during the reproductive age but no expression is detected after menopause. This correlates with the fact that PK1 expression is hormonally modulated, with oestrogen and progesterone increasing the expression of PK1 in the endometrium [39,43].

Further support for a role of PKs in reproductive function can be gleaned from PKR2 knockout mice, which show hypoplasia of the reproductive tract [28]. This phenotype has been explained by the lack of GnRH neurones in the hypothalamus of these animals. However, it is possible that the loss of a direct role of PKR2 in the reproductive tract contributes to the observed atrophy of these tissues. PK2 or PKR1 knockout mice do not show abnormalities in the reproductive tract [28,30].

## Potential role of PKs in pregnancy

### Implantation

PK1, but not PK2, PKR1 or PKR2, expression peaks during the midluteal ‘window of implantation’, with immunolocalization to endometrial glandular epithelium, stromal and endothelial cells, and myometrial vascular endothelium as well as smooth muscle [39,43,44].

Hyperaemia and endothelial leakage at the implantation site are one of the earliest signs of implantation [45,46]. It has been hypothesized that, by inducing fenestrae formation and increasing microvascular permeability, PK1 might be involved in effecting this hyperpermeability, thereby facilitating implantation [39,43]. More studies are required to evaluate whether PKs are involved in perimplantation spiral arteriole formation and recruitment of immune cells, including uterine natural killer cells, which have been shown to increase in number during the implantation window and early pregnancy [47].

### PKs and the fetoplacental unit

The expression of PK1 and PKR1 peaks in the trophoblast between 8–10 weeks of gestation, with PK1 being localized to the syncytiotrophoblast, minimal expression in the cytotrophoblast and no expression in the extravillous trophoblast [23]. This contrasts with VEGF and its receptors, which are predominantly expressed in the extravillous trophoblast and cytotrophoblast [23,48,49]. However, VEGF and PK1 are both expressed from six weeks of gestation in fetal Hofbauer cells in placental villous mesenchyme. PK2 and PKR2 are not expressed in first trimester trophoblasts, apart from between 8–10 weeks of gestation, when low levels of expression are detected. Expression of PKR2 in fetal vascular endothelial cells rises after 12 weeks of gestation [23]. Although expression of PK1 and PK2, the latter at considerably lower levels, has been demonstrated in third trimester placentae, neither cellular localization nor receptor expression has been studied [2,6,20,50]. Similarly, no studies have investigated the expression of PKs or their receptors in first trimester decidua or myometrium, or in the second trimester fetoplacental unit.

It has been proposed that PK1 and PKR1 have an important role in placentation, owing to their expression peaking during the crucial hypoxic period of placentation (8th–10th week) before establishment of the haemochorial circulation [23]. Hypoxic regulation of PK1 and PKR1 is supported by the presence of a hypoxia-inducible factor (HIF-1α) binding site in the promoter of both PK1 and PKR1. However, factors such as human chorionic gonadotropin [43] and progesterone [39], which are known to regulate PK1 expression, are also expressed during this period and they might therefore participate in PK1 regulation. The strong expression of PK1 by the syncytiotrophoblast, which is the endocrine component of the placenta, suggests that PK1 expression might be associated with a degree of trophoblast differentiation, and it has been hypothesized that PK1 might be a novel placental growth factor [23]. More studies are required to evaluate whether PKs are involved in fetal haematopoiesis, immune cell trafficking, maintenance of syncytial integrity and myometrial contractility.

## Role of PKs in pathologies of the reproductive tract

Several studies suggest that PKs have a role in pathological conditions of the reproductive tract. In the male, Samson *et al.* [35] demonstrated that PK1 is expressed in Leydig cell neoplasms. The increased microvasculature observed in these types of tumour compared with other types of testicular cancer, such as seminoma, suggest that PK1 has a role in Leydig cell tumour growth by promoting angiogenesis [35]. Also, PK1 is localized, at low levels, in glandular epithelial cells and hyperplastic benign prostate tissue, and increasing levels are detected in prostate cancer as the disease progresses [38]. These data suggest that PK1 could have an important role in Leydig cell tumours and prostate cancer, and could therefore be used as a marker for disease progression. In prostate cancer, increased mRNA expression of PK2 is also observed in malignant epithelial cells compared with normal epithelium, suggesting that both PKs might have a role in this pathology [38].

A direct role for PKs in cancers of the female reproductive tract has not been ascertained. In endometrial and ovarian carcinoma, it has been shown that PK1 expression is either absent or reduced compared with normal tissue [40,43]. However, a putative role for PK1 in the evolution of ovarian carcinoma has been suggested from the observation that this factor is expressed in non-tumour stromal cells and tumour-infiltrating T lymphocytes in this carcinoma [40].

Hyperplasia and hypervascularity of the stroma are key features in the development of polycystic ovarian syndrome (PCOS). The search for factors responsible for these features has identified strong expression of VEGF and PK1 in PCOS [21]. Strong correlation between PK1 expression and hyperplasia and angiogenesis has been reported, suggesting that PK1 could have a role in the development of this pathology [21]. Such a role is further supported by observations that adenoviral delivery of PKs to rat ovaries results in a phenotype similar to PCOS [2]. In addition, it has been suggested that PK1 could have a role, together with VEGF [51], in mediating the dysregulated vascular permeability that occurs in ovarian hyperstimulation syndrome [21].

In preeclampsia, the underlying pathophysiology remains unclear but the resulting placenta is relatively hypoxic, with evidence of chronic inflammatory and vaso-occlusive lesions. A study by Chung *et al.* [50] reported no difference in PK1 expression in third trimester placentae from either preeclamptic or normal pregnancies. However, increasing evidence suggests that the origin of preeclampsia is in the first trimester, with deficient adaptation between the maternal vascular system and fetoplacental unit. Changes in protein expression after disease onset are considered to be the consequence, rather than the cause, of its development. It has therefore been suggested that PK1 and PKR1 might have a role in the development of preeclampsia, given their temporal expression during the crucial hypoxic period of placentation during the first trimester [23].

## Summary

The diverse spectrum of established and potential regulatory functions of PKs in the nervous, immune, haematopoietic, vascular, gastrointestinal and reproductive systems suggest that they will be the targets of drug development for numerous pathologies. Within the reproductive system, they are expressed in the testis, prostate, ovary, uterus, placenta and neurones regulating GnRH neuronal development. The last of these is the only established function, and substantial experimental research is needed to complement the observational studies in this emerging arena, to establish functions in reproductive tissues. The development of immunoneutralizing antibodies and small-molecule antagonist and agonist analogues of these complex proteins will greatly enhance the elucidation of their functions, with the promise of establishing therapeutic interventions in reproductive pathologies. Such molecules will also aid in the dissection of the relative contribution of each of the PKs and their receptors to reproductive physiology and pathology. Moreover, future research aimed at the development of targeted and tissue-specific knockout of the genes encoding each of these proteins in mice will help to elucidate their specific role in reproductive function.

## Figures and Tables

**Table 1:** Binding affinities of PK1 and PK2 to PKR1 and PKR2, respectively^a^

	PKR1			PKR2		
	*K*_d_ (nM)	*K*_i_ (nM)	IC_50_ (nM)	*K*_d_ (nM)	*K*_i_ (nM)	IC_50_ (nM)
PK1	12.3 ± 4.2	66.3 ± 30.1	27.6 ± 8.2	1.8 ± 0.1	31.6 ± 13.7	52.2 ± 16.4
PK2	1.4 ± 0.5	5.1 ± 1.0	4.5 ± 0.8	2.0 ± 0.7	5.9 ± 1.3	6.4 ± 1.3

Abbreviations: *K*_d_, dissociation constant; *K*_i_, inhibitory constant; IC_50_, inhibitory concentration 50%.

a Values reported by Lin, D.C. *et al.*[14], Soga, T. *et al.*[16] and Chen, J. *et al.*[54].
